# Crystal structure and Hirshfeld analysis of 2-[bis­(1-methyl-1*H*-indol-3-yl)meth­yl]benzoic acid

**DOI:** 10.1107/S2056989018014160

**Published:** 2018-10-16

**Authors:** Suhaila Sapari, Sheryn Wong, Mohammad Fadzlee Ngatiman, Huda Misral, Siti Aishah Hasbullah

**Affiliations:** a Centre of Advanced Materials and Renewable Resources, Faculty of Science and Technology, National University of Malaysia, 43600 UKM Bangi, Selangor, Malaysia; b Center for Research and Instrumentation Management, Universiti Kebangsaan Malaysia, 43600 UKM Bangi, Selangor, Malaysia

**Keywords:** crystal structure, indole derivatives, benzoic acid

## Abstract

In the title compound, C_26_H_22_N_2_O_2_, the dihedral angles between the 1-methyl­indole units (*A* and *B*) and the benzoic acid moiety (*C*) are *A*/*B* = 64.87 (7), *A*/*C* = 80.92 (8) and *B*/*C* = 75.05 (8)°. An intra­molecular C—H⋯O inter­action arising from the methyne group helps to establish the conformation. In the crystal, *R*_2_^2^(8) carb­oxy­lic acid inversion dimers linked by pairs of O—H⋯O hydrogen bonds are observed. A Hirshfeld surface analysis shows that the greatest contributions are from H⋯H, C⋯H/H⋯C and O⋯H/H⋯O contacts (percentage values = 54.6%, 29.6% and 10.1%, respectively).

## Abstract 2

In the title compound, the dihedral angles between the 1-methyl indole units (*A* and *B*) and the benzoic acid moiety (*C*) are *A*/*B* = 64.87 (7), *A*/*C* = 80.92 (8) and *B*/*C* = 75.05 (8)°. An intra­molecular C—H⋯O inter­action arising from the methyne group helps to establish the conformation. In the crystal, 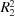
(8) carb­oxy­lic acid inversion dimers linked by pairs of O—H⋯O hydrogen bonds are observed.

## Chemical context   

Bisindolyl methane and its derivatives are relatively easy to synthesize and show a broad spectrum of potential biological activities: for example, bis­(indol­yl)imidazole shows anti­plasmodial activity towards *plasmodium falciparum* (Alvarado *et al.*, 2013 ▸). Furthermore, they also have good potential as anti­bacterial (Imran *et al.*, 2014 ▸; Challa *et al.*, 2017 ▸), anti­leishmanial (Bharate *et al.*, 2013 ▸), anti­tumor (Carbone *et al.*, 2013 ▸), anti­platelet (Grumel *et al.*, 2002 ▸) and anti­cancer (Guo *et al.*, 2010 ▸; Jamsheena *et al.*, 2016 ▸) agents. Oxidized bis­(indol­yl)methanes containing an acidic hydrogen-bond-donor group and a basic hydrogen-bond-acceptor group can act as selective colorimetric sensors for either F^−^ or HSO_4_^−^ in an aprotic solvent (He *et al.*, 2006 ▸). Aryl­furyl-bis­(indol­yl)methanes have selective chromogenic and fluoro­genic ratiometric receptors for the mercury ion in aqueous solution (Batista *et al.*, 2014 ▸). As part of our studies in this area, we now report the acid-catalysed condensation reaction between carb­oxy benzaldehyde and indole to generate the title compound.
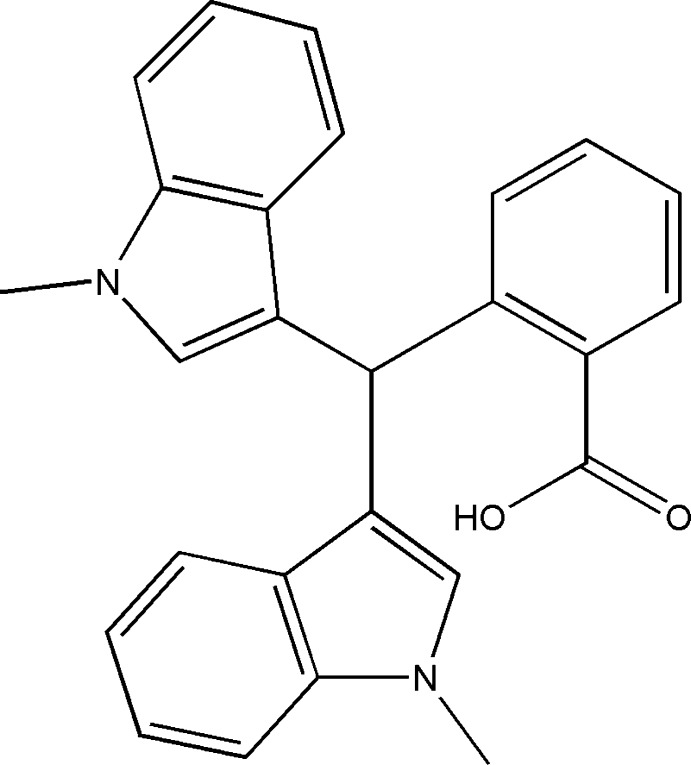


## Structural commentary   

**Figure 1:**
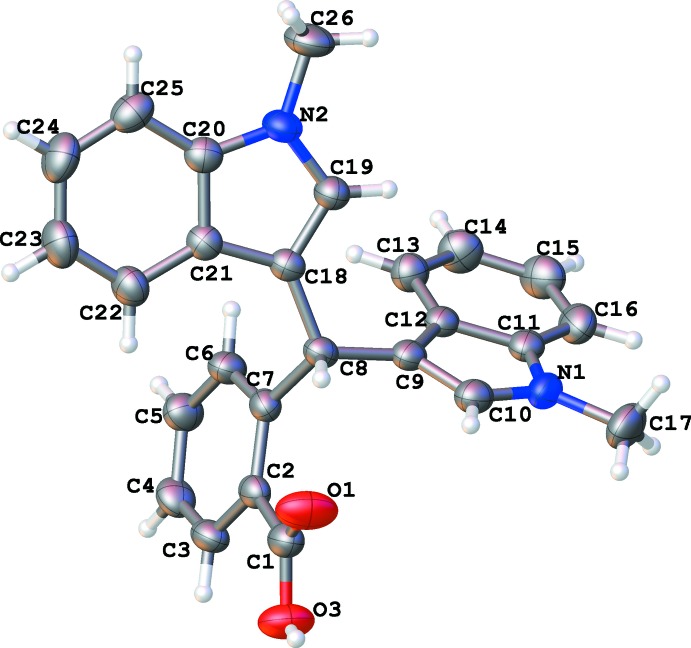
The mol­ecular structure of the title compound with displacement ellipsoids drawn at the 50% probability level.

The title compound (Fig. 1 ▸) crystallizes in the triclinic system with space group *P* 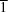 and *Z* = 2. The mol­ecule consists of two methyl­ated indole ring systems [C8–C17/N1 (*A*) and C18–C26/N2 (*B*)] and a benzoic acid [C1–C7 (*C*)] system linked *via* the tertiary C8 atom, with dihedral angles between them of *A*/*B* = 64.87 (7), *A*/*C* = 80.92 (8) and *B*/*C* = 75.05 (8)°. Significant torsion angles include C7—C8—C9—C12 [67.3 (3)] and C7—C8—C18—C21 [50.2 (3)°]. An intra­molecular C8—H8⋯O1 hydrogen bond (Table 1 ▸) may help to establish the conformation.

## Supra­molecular features   

**Figure 2:**
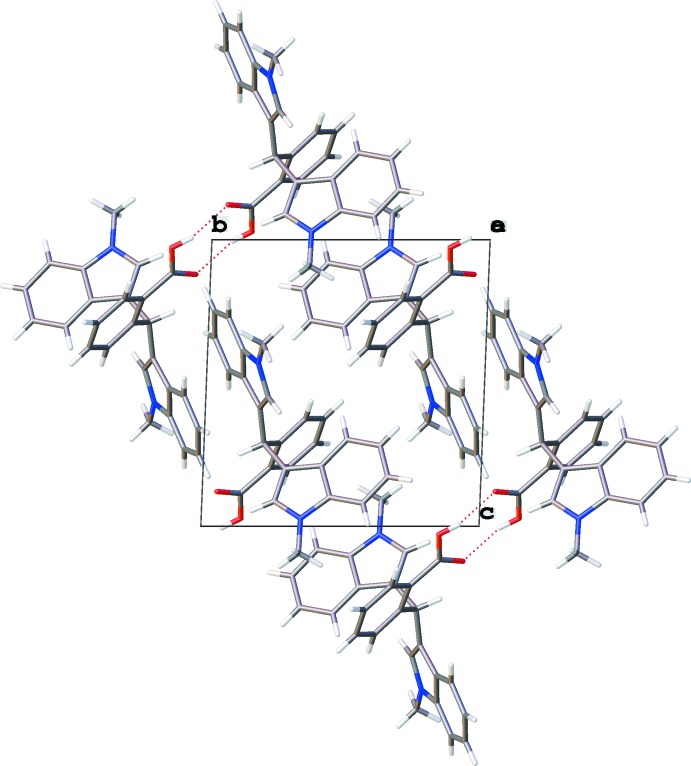
Crystal packing of the title compound viewed down [100] showing inversion dimers linked by pairs of O—H⋯O hydrogen bonds (dashed lines; Table 1 ▸).

In the crystal of the title compound, neighbouring mol­ecules are connected into dimers with an 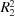
(8) graph-set motif *via* pairwise O3—H3*O*⋯O1 hydrogen bonds (Table 1 ▸, Fig. 2 ▸).

## Hirshfeld surface analysis   

**Figure 3:**
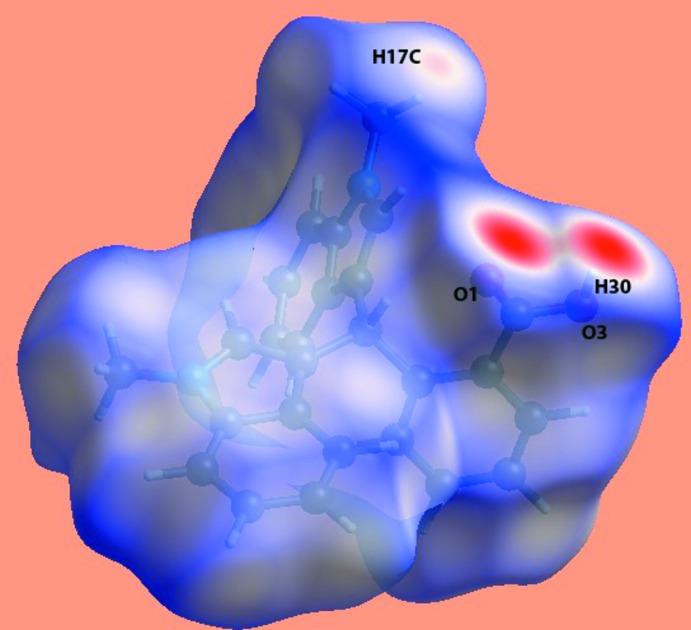
View of the Hirshfeld surface of the title compound mapped over *d*_norm_ in the range −0.68 to +1.45 au.

**Figure 4:**
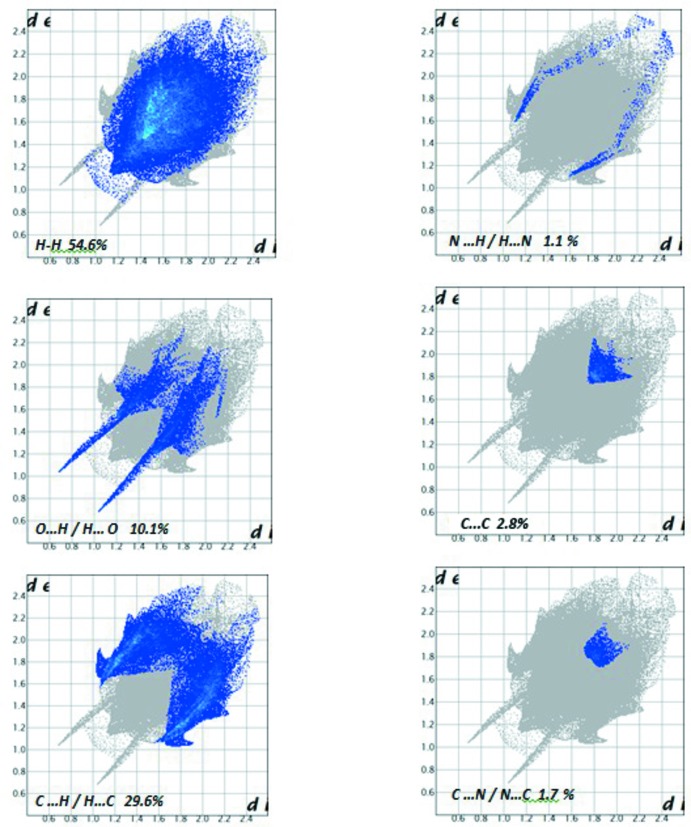
Two-dimensional fingerprint plots of the title compound delineated into H⋯H, O⋯H/H⋯O, C⋯H/H⋯C, N⋯H/H⋯N, C⋯C, C⋯N/N⋯C contacts.

The Hirshfeld surface and fingerprint (FP) plots for the title compound were generated using *CrystalExplorer17* (McKinnon *et al.*, 2007 ▸). A view of the Hirshfeld surface mapped over *d*_norm_ is shown in Fig. 3 ▸. The intense red spots near the O1-carbonyl and H30-benzoic acid atoms indicate the short inter­atomic O⋯H/H⋯O contacts relating to the hydrogen bond given in Table 1 ▸. The two-dimensional fingerprint plots for the H⋯H, O⋯H/H⋯O, C⋯H/H⋯C, N⋯H/H⋯N, C⋯C and C⋯N/C⋯N contacts are illustrated in Fig. 4 ▸. The percentage contributions from the different inter­atomic contact to the Hirshfeld surface are summarized in Table 2 ▸. The fingerprint plot for the H⋯H contacts, which make the largest contribution to the Hirshfeld surface (54.6%), has a broad appearance with a single tip at *d*_e_ + *d*_i_ = 2.2 Å. The FP plot for the O⋯H/H⋯O (10.1%) contacts has prominent ‘forceps-like’ tips at *d*_e_ + *d*_i_ = 1.7 Å, whereas that for C⋯H/H⋯C contacts (29.6%) shows two pairs of adjacent peaks with *d*_e_ + *d*_i_ = 2.6 Å. The other remaining inter­atomic contacts, which make a small percentage contribution, have a negligible effect on the packing.

## Database survey   

A search of the Cambridge Structural Database (Groom *et al.*, 2016 ▸) revealed only seven structures of bis­(indole-3-yl) deriv­atives. These include 3,5-bis­(indol-3-yl)-1,2,4-triazin-6(1*H*,6*H*)-one methanol solvate (FOLSOP) and 3,6-bis­(indol-3-yl)-1,2,4-triazin-4(1*H*,4*H*)-one di­methyl­formamide solvate (FOLTAC; Garg & Stoltz, 2005 ▸), bis­(indol-3-yl)(*p*-tol­yl)methane (HODROH; Krishna *et al.*, 1999 ▸), 1,1-bis­(indol-3-yl)-1-phenyl­ethane (MEDJEK; Ganesan *et al.*, 2000 ▸), *cyclo*-*N*,*N*′-(α,α′-*p*-xyl­yl)bis­(indol-3-yl)-*N*-methyl­male­imide (UJALOG), *cyclo*-*N*,*N*′-(α,α′-*m*-xyl­yl)bis­(indol-3-yl)-*N*-methyl­male­imide (UJALUM) and *cyclo*-*N*,*N*′-[1,11-(3,6,9-trioxaundec­yl)]bis(indol-3-yl)-*N*-methyl­male­imide (UJAMAT; Mandl *et al.*, 2003 ▸). Two of these entries (MEDJEK and HODROH) are closely related to the title compound. Two of these entries (MEDJEK and HODROH) are closely related to the title compound with dihedral angles between the 1-methyl indole units of 63.4 (2) and 73.06 (19)° for the two independent mol­ecules in MEDJEK and of 80.8 (1)° in HODROH [64.87 (7)° in the title compound]. In another related compound 4-[bis­(1*H*-indol-3-yl)meth­yl]benzo­nitrile (Deng *et al.*, 2011 ▸), the dihedral angle is 72.08 (6)°.

## Synthesis and crystallization   

Equimolar amounts of 2-carb­oxy­benzaldehyde (3.0 mmol) and 1-methyl­indole (3.0 mmol) was mixed in a reaction vessel. A few drops of anhydrous acetic acid was added and the mixture was then irradiated in a domestic microwave oven at 100 W for 5 min. The crude product obtained was purified by recrystallization from an acetone–EtOH solvent mixture (*v*:*v* = 1:2) to give the pure product in 13.3% yield. IR (ATR, υ_max_/cm^−1^): 3058, 2930 (*broad*, O—H), 1676 (C=O), 1473 (C=C), 1331–1067 (C—O, C—N), 731. ^1^H NMR (400 MHz, DMSO-*d_6_*) δ (ppm): 3.67 (*s*, 6H, 2 × N-C*H_3_*), 6.70 (*s*, 2H, 2 × H), 6.91 (*t*, 2H, 2 × ArH), 6.99 (*s*, 1H, H), 7.11 (*t*, 2H, 2 × ArH), 7.25–7.30 (*m*, 3H, *J* = 7.6, 6.6, 2.2 Hz, ArH and 2 × ArH), 7.35–7.41 (*m*, 4H,*J* = 8.0, 5.6, 1.2 Hz, ArH and 2 × ArH), 7.77 (*d*, 1H, *J* = 8.0 Hz, ArH) (the OH signal cannot be seen in the ^1^H NMR sprectrum and hence there are only 21 H atoms in the integration peaks). ^13^C NMR (101 MHz, DMSO-*d_6_*) δ (ppm): 32.7, 34.5, 110.1, 117.9, 118.9, 119.5, 121.6, 126.4, 127.4, 128.6, 130.0, 130.1, 131.3, 131.6, 137.4, 145.2, 170.1.

## Refinement   

Crystal data, data collection and structure refinement details are summarized in Table 3 ▸. The hy­droxy H atom was freely refined. C-bound H atoms were positioned geometrically and refined using a riding model with C—H = 0.93–0.96 and *U*_iso_(H) = 1.2–1.5*U*_eq_(C).

## Supplementary Material

Crystal structure: contains datablock(s) I. DOI: 10.1107/S2056989018014160/hb7773sup1.cif

Structure factors: contains datablock(s) I. DOI: 10.1107/S2056989018014160/hb7773Isup3.hkl

Supporting information file. DOI: 10.1107/S2056989018014160/hb7773Isup4.cml

Supplementary figures. DOI: 10.1107/S2056989018014160/hb7773sup5.pdf

CCDC reference: 1871874

Additional supporting information:  crystallographic information; 3D view; checkCIF report

## Figures and Tables

**Table 1:** Hydrogen-bond geometry (Å, °)

*D*—H⋯*A*	*D*—H	H⋯*A*	*D*⋯*A*	*D*—H⋯*A*
O3—H3*O*⋯O1^i^	0.82 (4)	1.89 (5)	2.679 (3)	163 (6)
C8—H8⋯O1	0.98	2.20	2.945 (4)	132

Symmetry code: (i) 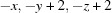.

**Table 2:** Percentage contributions of inter­atomic contacts to the Hirshfield surface of the title compound

Contact	Percentage contribution
H⋯H	54.6
O⋯H/H⋯O	10.1
C⋯H/H⋯C	29.6
N⋯H/H⋯N	1.1
C⋯N/C⋯N	1.7
C⋯C	2.8

**Table 3:** Experimental details

Crystal data	
Chemical formula	C_26_H_22_N_2_O_2_
*M*_r_	394.45
Crystal system, space group	Triclinic, *P* 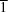
Temperature (K)	293
*a*, *b*, *c* (Å)	8.654 (5), 10.923 (6), 10.964 (5)
α, β, γ (°)	85.85 (2), 82.38 (2), 74.57 (3)
*V* (Å^3^)	989.4 (9)
*Z*	2
Radiation type	Mo *K*α
μ (mm^−1^)	0.08
Crystal size (mm)	0.55 × 0.39 × 0.30

Data collection	
Diffractometer	Bruker PHOTON 100 CMOS
Absorption correction	Multi-scan (*SADABS*; Bruker, 2016 ▸)
*T*_min_, *T*_max_	0.548, 0.746
No. of measured, independent and observed [*I* > 2σ(*I*)] reflections	37627, 4929, 3077
*R*_int_	0.101
(sin θ/λ)_max_ (Å^−1^)	0.669

Refinement	
*R*[*F* ^2^ > 2σ(*F* ^2^)], *wR*(*F* ^2^), *S*	0.069, 0.190, 1.03
No. of reflections	4929
No. of parameters	277
No. of restraints	1
H-atom treatment	H atoms treated by a mixture of independent and constrained refinement
Δρ_max_, Δρ_min_ (e Å^−3^)	0.72, −0.35

Computer programs: *APEX3* and *SAINT* (Bruker, 2016 ▸), *SHELXT2014/5* (Sheldrick, 2015*a* ▸), *SHELXL* (Sheldrick, 2015*b* ▸), *shelXle* (Hübschle *et al.*, 2011 ▸), *SHELXTL* (Sheldrick, 2008 ▸), *PLATON* (Spek, 2009 ▸), *OLEX2* (Dolomanov *et al.*, 2009 ▸) and *publCIF* (Westrip, 2010 ▸).

## supplementary crystallographic information

### Crystal data

**Table:**

C_26_H_22_N_2_O_2_	*Z* = 2
*M**_r_* = 394.45	*F*(000) = 416
Triclinic, *P*1	*D*_x_ = 1.324 Mg m^−^^3^
*a* = 8.654 (5) Å	Mo *K*α radiation, λ = 0.71076 Å
*b* = 10.923 (6) Å	Cell parameters from 8410 reflections
*c* = 10.964 (5) Å	θ = 2.9–27.3°
α = 85.85 (2)°	µ = 0.08 mm^−^^1^
β = 82.38 (2)°	*T* = 293 K
γ = 74.57 (3)°	Block, colourless
*V* = 989.4 (9) Å^3^	0.55 × 0.39 × 0.30 mm

### Data collection

**Table:**

Bruker PHOTON 100 CMOS diffractometer	3077 reflections with *I* > 2σ(*I*)
Radiation source: fine-focus sealed tube	*R*_int_ = 0.101
φ and ω scans	θ_max_ = 28.4°, θ_min_ = 2.9°
Absorption correction: multi-scan (SADABS; Bruker, 2016)	*h* = −11→11
*T*_min_ = 0.548, *T*_max_ = 0.746	*k* = −14→14
37627 measured reflections	*l* = −14→14
4929 independent reflections	

### Refinement

**Table:**

Refinement on *F*^2^	Primary atom site location: structure-invariant direct methods
Least-squares matrix: full	Hydrogen site location: mixed
*R*[*F*^2^ > 2σ(*F*^2^)] = 0.069	H atoms treated by a mixture of independent and constrained refinement
*wR*(*F*^2^) = 0.190	*w* = 1/[σ^2^(*F*_o_^2^) + (0.0831*P*)^2^ + 0.5551*P*] where *P* = (*F*_o_^2^ + 2*F*_c_^2^)/3
*S* = 1.03	(Δ/σ)_max_ < 0.001
4929 reflections	Δρ_max_ = 0.72 e Å^−^^3^
277 parameters	Δρ_min_ = −0.35 e Å^−^^3^
1 restraint	

### Special details

**Table:**

Geometry. All esds (except the esd in the dihedral angle between two l.s. planes) are estimated using the full covariance matrix. The cell esds are taken into account individually in the estimation of esds in distances, angles and torsion angles; correlations between esds in cell parameters are only used when they are defined by crystal symmetry. An approximate (isotropic) treatment of cell esds is used for estimating esds involving l.s. planes.
Refinement. Refinement of *F*^2^ against ALL reflections. The weighted *R*-factor *wR* and goodness of fit *S* are based on *F*^2^, conventional *R*-factors *R* are based on *F*, with *F* set to zero for negative *F*^2^. The threshold expression of *F*^2^ > σ(*F*^2^) is used only for calculating *R*-factors(gt) *etc*. and is not relevant to the choice of reflections for refinement. *R*-factors based on *F*^2^ are statistically about twice as large as those based on *F*, and *R*- factors based on ALL data will be even larger.

### Fractional atomic coordinates and isotropic or equivalent isotropic displacement parameters (Å^2^)

**Table:**

	*x*	*y*	*z*	*U*_iso_*/*U*_eq_	
O1	0.1286 (3)	0.9468 (2)	0.8786 (2)	0.0672 (6)	
C1	0.0635 (3)	0.8626 (2)	0.8846 (2)	0.0418 (6)	
H3O	−0.070 (8)	0.925 (3)	1.005 (6)	0.20 (3)*	
C26	0.8219 (3)	0.8016 (3)	0.3643 (3)	0.0575 (7)	
H26A	0.840826	0.738582	0.303606	0.086*	
H26B	0.901263	0.775487	0.420976	0.086*	
H26C	0.829479	0.881343	0.324457	0.086*	
C25	0.4893 (3)	0.9294 (2)	0.2705 (2)	0.0465 (6)	
H25	0.573439	0.932437	0.209051	0.056*	
C24	0.3315 (4)	0.9808 (3)	0.2504 (3)	0.0556 (7)	
H24	0.308350	1.019279	0.174135	0.067*	
N1	0.6152 (2)	0.6429 (2)	0.97202 (18)	0.0427 (5)	
N2	0.6626 (2)	0.81578 (19)	0.43055 (18)	0.0403 (5)	
C2	0.1002 (3)	0.7587 (2)	0.7937 (2)	0.0346 (5)	
C14	0.7258 (3)	0.3672 (3)	0.7182 (3)	0.0517 (7)	
H14	0.748386	0.307509	0.657587	0.062*	
C3	−0.0168 (3)	0.6924 (2)	0.7922 (2)	0.0420 (6)	
H3	−0.109288	0.711578	0.848612	0.050*	
O3	−0.0538 (3)	0.8571 (2)	0.97210 (19)	0.0631 (6)	
C4	0.0012 (3)	0.5995 (2)	0.7097 (2)	0.0456 (6)	
H4	−0.076746	0.554773	0.711828	0.055*	
C5	0.1353 (3)	0.5732 (3)	0.6238 (2)	0.0452 (6)	
H5	0.147565	0.512118	0.565909	0.054*	
C6	0.2518 (3)	0.6386 (2)	0.6243 (2)	0.0419 (6)	
H6	0.341532	0.621037	0.565193	0.050*	
C7	0.2402 (3)	0.7292 (2)	0.7094 (2)	0.0341 (5)	
C21	0.3939 (3)	0.8673 (2)	0.4803 (2)	0.0339 (5)	
C8	0.3842 (3)	0.7849 (2)	0.7119 (2)	0.0332 (5)	
H8	0.341561	0.869135	0.746666	0.040*	
C22	0.2345 (3)	0.9212 (2)	0.4559 (2)	0.0436 (6)	
H22	0.148793	0.919612	0.516422	0.052*	
C9	0.4939 (3)	0.7048 (2)	0.7995 (2)	0.0347 (5)	
C23	0.2062 (3)	0.9762 (3)	0.3419 (3)	0.0543 (7)	
H23	0.100327	1.011219	0.325564	0.065*	
C10	0.5135 (3)	0.7397 (2)	0.9123 (2)	0.0414 (6)	
H10	0.464125	0.819036	0.944587	0.050*	
C11	0.6628 (3)	0.5408 (2)	0.8979 (2)	0.0368 (5)	
C13	0.6259 (3)	0.4862 (2)	0.6972 (2)	0.0421 (6)	
H13	0.582726	0.506790	0.622801	0.050*	
C12	0.5903 (3)	0.5755 (2)	0.7882 (2)	0.0339 (5)	
C19	0.6294 (3)	0.7755 (2)	0.5505 (2)	0.0371 (5)	
H19	0.707140	0.734204	0.601145	0.044*	
C15	0.7928 (3)	0.3347 (3)	0.8272 (3)	0.0542 (7)	
H15	0.858248	0.253282	0.839323	0.065*	
C17	0.6653 (4)	0.6456 (3)	1.0926 (2)	0.0616 (8)	
H17A	0.769095	0.663417	1.083578	0.092*	
H17B	0.672004	0.564573	1.134905	0.092*	
H17C	0.587916	0.710521	1.139210	0.092*	
C16	0.7642 (3)	0.4213 (3)	0.9186 (2)	0.0493 (6)	
H16	0.811197	0.400285	0.991351	0.059*	
C20	0.5193 (3)	0.8728 (2)	0.3856 (2)	0.0363 (5)	
C18	0.4677 (3)	0.8041 (2)	0.5851 (2)	0.0322 (5)	

### Atomic displacement parameters (Å^2^)

**Table:**

	*U*^11^	*U*^22^	*U*^33^	*U*^12^	*U*^13^	*U*^23^
O1	0.0693 (14)	0.0618 (13)	0.0698 (14)	−0.0252 (11)	0.0249 (11)	−0.0267 (11)
C1	0.0343 (12)	0.0498 (15)	0.0381 (13)	−0.0080 (11)	−0.0023 (10)	0.0048 (11)
C26	0.0394 (14)	0.0683 (19)	0.0607 (18)	−0.0173 (13)	0.0129 (12)	0.0022 (14)
C25	0.0609 (16)	0.0423 (13)	0.0386 (13)	−0.0200 (12)	−0.0030 (12)	0.0028 (11)
C24	0.0698 (19)	0.0517 (16)	0.0477 (16)	−0.0171 (14)	−0.0213 (14)	0.0135 (12)
N1	0.0414 (11)	0.0582 (13)	0.0315 (10)	−0.0179 (10)	−0.0058 (8)	−0.0007 (9)
N2	0.0317 (10)	0.0449 (11)	0.0427 (11)	−0.0119 (8)	0.0025 (8)	0.0025 (9)
C2	0.0281 (10)	0.0387 (12)	0.0344 (11)	−0.0053 (9)	−0.0052 (9)	0.0066 (9)
C14	0.0551 (16)	0.0435 (14)	0.0540 (16)	−0.0087 (12)	−0.0038 (13)	−0.0073 (12)
C3	0.0290 (11)	0.0503 (14)	0.0440 (13)	−0.0097 (10)	0.0006 (10)	0.0045 (11)
O3	0.0614 (13)	0.0700 (14)	0.0543 (12)	−0.0209 (11)	0.0201 (10)	−0.0137 (11)
C4	0.0379 (13)	0.0517 (15)	0.0535 (15)	−0.0237 (11)	−0.0074 (11)	0.0056 (12)
C5	0.0428 (13)	0.0518 (15)	0.0448 (14)	−0.0182 (11)	−0.0032 (11)	−0.0067 (11)
C6	0.0327 (12)	0.0542 (15)	0.0394 (13)	−0.0144 (11)	0.0013 (10)	−0.0040 (11)
C7	0.0287 (10)	0.0411 (12)	0.0329 (11)	−0.0104 (9)	−0.0055 (9)	0.0050 (9)
C21	0.0348 (11)	0.0290 (11)	0.0380 (12)	−0.0089 (9)	−0.0040 (9)	−0.0002 (9)
C8	0.0275 (10)	0.0372 (12)	0.0351 (12)	−0.0096 (9)	−0.0004 (9)	−0.0034 (9)
C22	0.0373 (12)	0.0411 (13)	0.0495 (15)	−0.0047 (10)	−0.0080 (11)	0.0016 (11)
C9	0.0292 (11)	0.0442 (13)	0.0326 (11)	−0.0147 (9)	0.0001 (9)	−0.0016 (9)
C23	0.0486 (15)	0.0500 (16)	0.0623 (18)	−0.0049 (12)	−0.0209 (13)	0.0071 (13)
C10	0.0375 (12)	0.0496 (14)	0.0383 (13)	−0.0154 (11)	0.0003 (10)	−0.0038 (11)
C11	0.0326 (11)	0.0463 (13)	0.0341 (12)	−0.0174 (10)	−0.0008 (9)	0.0023 (10)
C13	0.0420 (13)	0.0439 (14)	0.0416 (13)	−0.0127 (11)	−0.0060 (10)	−0.0024 (11)
C12	0.0290 (10)	0.0422 (12)	0.0323 (11)	−0.0144 (9)	−0.0001 (9)	−0.0003 (9)
C19	0.0323 (11)	0.0415 (13)	0.0369 (12)	−0.0106 (10)	−0.0022 (9)	0.0016 (10)
C15	0.0528 (16)	0.0438 (15)	0.0589 (17)	−0.0042 (12)	−0.0037 (13)	0.0072 (13)
C17	0.0698 (19)	0.087 (2)	0.0350 (14)	−0.0299 (17)	−0.0130 (13)	−0.0032 (14)
C16	0.0462 (14)	0.0571 (16)	0.0433 (14)	−0.0139 (12)	−0.0085 (11)	0.0141 (12)
C20	0.0381 (12)	0.0321 (11)	0.0399 (12)	−0.0125 (9)	−0.0021 (10)	−0.0017 (9)
C18	0.0293 (10)	0.0304 (11)	0.0363 (12)	−0.0082 (9)	−0.0009 (9)	−0.0018 (9)

### Geometric parameters (Å, º)

**Table:**

O1—C1	1.193 (3)	C5—H5	0.9300
C1—O3	1.311 (3)	C6—C7	1.384 (3)
C1—C2	1.507 (4)	C6—H6	0.9300
C26—N2	1.445 (3)	C7—C8	1.529 (3)
C26—H26A	0.9600	C21—C22	1.402 (3)
C26—H26B	0.9600	C21—C20	1.410 (3)
C26—H26C	0.9600	C21—C18	1.431 (3)
C25—C24	1.371 (4)	C8—C9	1.507 (3)
C25—C20	1.389 (3)	C8—C18	1.507 (3)
C25—H25	0.9300	C8—H8	0.9800
C24—C23	1.385 (4)	C22—C23	1.370 (4)
C24—H24	0.9300	C22—H22	0.9300
N1—C11	1.368 (3)	C9—C10	1.365 (3)
N1—C10	1.371 (3)	C9—C12	1.441 (3)
N1—C17	1.449 (3)	C23—H23	0.9300
N2—C20	1.370 (3)	C10—H10	0.9300
N2—C19	1.375 (3)	C11—C16	1.388 (4)
C2—C3	1.395 (3)	C11—C12	1.410 (3)
C2—C7	1.402 (3)	C13—C12	1.394 (3)
C14—C13	1.379 (4)	C13—H13	0.9300
C14—C15	1.380 (4)	C19—C18	1.358 (3)
C14—H14	0.9300	C19—H19	0.9300
C3—C4	1.373 (4)	C15—C16	1.380 (4)
C3—H3	0.9300	C15—H15	0.9300
O3—H3O	0.820 (10)	C17—H17A	0.9600
C4—C5	1.374 (3)	C17—H17B	0.9600
C4—H4	0.9300	C17—H17C	0.9600
C5—C6	1.383 (3)	C16—H16	0.9300
			
O1—C1—O3	120.9 (2)	C9—C8—C7	108.83 (18)
O1—C1—C2	124.9 (2)	C18—C8—C7	112.70 (18)
O3—C1—C2	114.1 (2)	C9—C8—H8	106.9
N2—C26—H26A	109.5	C18—C8—H8	106.9
N2—C26—H26B	109.5	C7—C8—H8	106.9
H26A—C26—H26B	109.5	C23—C22—C21	119.3 (2)
N2—C26—H26C	109.5	C23—C22—H22	120.3
H26A—C26—H26C	109.5	C21—C22—H22	120.3
H26B—C26—H26C	109.5	C10—C9—C12	105.5 (2)
C24—C25—C20	117.9 (2)	C10—C9—C8	125.6 (2)
C24—C25—H25	121.1	C12—C9—C8	128.7 (2)
C20—C25—H25	121.1	C22—C23—C24	121.6 (3)
C25—C24—C23	121.0 (3)	C22—C23—H23	119.2
C25—C24—H24	119.5	C24—C23—H23	119.2
C23—C24—H24	119.5	C9—C10—N1	111.3 (2)
C11—N1—C10	108.1 (2)	C9—C10—H10	124.4
C11—N1—C17	125.0 (2)	N1—C10—H10	124.4
C10—N1—C17	126.9 (2)	N1—C11—C16	129.4 (2)
C20—N2—C19	108.30 (19)	N1—C11—C12	108.1 (2)
C20—N2—C26	126.1 (2)	C16—C11—C12	122.5 (2)
C19—N2—C26	125.6 (2)	C14—C13—C12	119.5 (2)
C3—C2—C7	119.2 (2)	C14—C13—H13	120.2
C3—C2—C1	116.8 (2)	C12—C13—H13	120.2
C7—C2—C1	123.9 (2)	C13—C12—C11	118.0 (2)
C13—C14—C15	121.4 (3)	C13—C12—C9	135.0 (2)
C13—C14—H14	119.3	C11—C12—C9	107.0 (2)
C15—C14—H14	119.3	C18—C19—N2	110.8 (2)
C4—C3—C2	121.6 (2)	C18—C19—H19	124.6
C4—C3—H3	119.2	N2—C19—H19	124.6
C2—C3—H3	119.2	C14—C15—C16	121.0 (2)
C1—O3—H3O	102 (5)	C14—C15—H15	119.5
C3—C4—C5	119.5 (2)	C16—C15—H15	119.5
C3—C4—H4	120.2	N1—C17—H17A	109.5
C5—C4—H4	120.2	N1—C17—H17B	109.5
C4—C5—C6	119.2 (2)	H17A—C17—H17B	109.5
C4—C5—H5	120.4	N1—C17—H17C	109.5
C6—C5—H5	120.4	H17A—C17—H17C	109.5
C5—C6—C7	122.6 (2)	H17B—C17—H17C	109.5
C5—C6—H6	118.7	C15—C16—C11	117.6 (2)
C7—C6—H6	118.7	C15—C16—H16	121.2
C6—C7—C2	117.7 (2)	C11—C16—H16	121.2
C6—C7—C8	118.5 (2)	N2—C20—C25	130.2 (2)
C2—C7—C8	123.7 (2)	N2—C20—C21	107.6 (2)
C22—C21—C20	118.0 (2)	C25—C20—C21	122.2 (2)
C22—C21—C18	134.8 (2)	C19—C18—C21	106.1 (2)
C20—C21—C18	107.2 (2)	C19—C18—C8	126.7 (2)
C9—C8—C18	114.13 (18)	C21—C18—C8	126.96 (19)
			
C20—C25—C24—C23	−0.1 (4)	C14—C13—C12—C11	1.9 (3)
O1—C1—C2—C3	−161.0 (3)	C14—C13—C12—C9	−179.8 (2)
O3—C1—C2—C3	16.6 (3)	N1—C11—C12—C13	178.30 (19)
O1—C1—C2—C7	16.6 (4)	C16—C11—C12—C13	−1.4 (3)
O3—C1—C2—C7	−165.8 (2)	N1—C11—C12—C9	−0.5 (2)
C7—C2—C3—C4	−0.5 (3)	C16—C11—C12—C9	179.8 (2)
C1—C2—C3—C4	177.2 (2)	C10—C9—C12—C13	−178.4 (2)
C2—C3—C4—C5	−1.9 (4)	C8—C9—C12—C13	5.4 (4)
C3—C4—C5—C6	1.7 (4)	C10—C9—C12—C11	0.1 (2)
C4—C5—C6—C7	0.8 (4)	C8—C9—C12—C11	−176.1 (2)
C5—C6—C7—C2	−3.1 (4)	C20—N2—C19—C18	0.3 (3)
C5—C6—C7—C8	172.9 (2)	C26—N2—C19—C18	179.6 (2)
C3—C2—C7—C6	2.9 (3)	C13—C14—C15—C16	−1.0 (4)
C1—C2—C7—C6	−174.7 (2)	C14—C15—C16—C11	1.5 (4)
C3—C2—C7—C8	−172.9 (2)	N1—C11—C16—C15	−179.9 (2)
C1—C2—C7—C8	9.5 (3)	C12—C11—C16—C15	−0.2 (4)
C6—C7—C8—C9	−90.0 (2)	C19—N2—C20—C25	179.3 (2)
C2—C7—C8—C9	85.8 (3)	C26—N2—C20—C25	0.1 (4)
C6—C7—C8—C18	37.6 (3)	C19—N2—C20—C21	−0.2 (3)
C2—C7—C8—C18	−146.6 (2)	C26—N2—C20—C21	−179.4 (2)
C20—C21—C22—C23	0.4 (3)	C24—C25—C20—N2	−179.5 (2)
C18—C21—C22—C23	179.7 (3)	C24—C25—C20—C21	−0.1 (4)
C18—C8—C9—C10	125.1 (2)	C22—C21—C20—N2	179.5 (2)
C7—C8—C9—C10	−108.1 (2)	C18—C21—C20—N2	0.0 (2)
C18—C8—C9—C12	−59.5 (3)	C22—C21—C20—C25	0.0 (3)
C7—C8—C9—C12	67.3 (3)	C18—C21—C20—C25	−179.5 (2)
C21—C22—C23—C24	−0.6 (4)	N2—C19—C18—C21	−0.3 (3)
C25—C24—C23—C22	0.4 (4)	N2—C19—C18—C8	−174.8 (2)
C12—C9—C10—N1	0.4 (3)	C22—C21—C18—C19	−179.2 (3)
C8—C9—C10—N1	176.70 (19)	C20—C21—C18—C19	0.2 (2)
C11—N1—C10—C9	−0.7 (3)	C22—C21—C18—C8	−4.7 (4)
C17—N1—C10—C9	179.4 (2)	C20—C21—C18—C8	174.7 (2)
C10—N1—C11—C16	−179.6 (2)	C9—C8—C18—C19	−11.6 (3)
C17—N1—C11—C16	0.3 (4)	C7—C8—C18—C19	−136.4 (2)
C10—N1—C11—C12	0.7 (2)	C9—C8—C18—C21	175.0 (2)
C17—N1—C11—C12	−179.4 (2)	C7—C8—C18—C21	50.2 (3)
C15—C14—C13—C12	−0.7 (4)		

### Hydrogen-bond geometry (Å, º)

**Table:**

*D*—H···*A*	*D*—H	H···*A*	*D*···*A*	*D*—H···*A*
O3—H3*O*···O1^i^	0.82 (4)	1.89 (5)	2.679 (3)	163 (6)
C8—H8···O1	0.98	2.20	2.945 (4)	132

Symmetry code: (i) −*x*, −*y*+2, −*z*+2.

### Percentage contributions of interatomic contacts to the Hirshfeld surface for (I)

**Table:**

Contact	Percentage contribution
H ··· H	54.6
O ··· H / H ··· O	10.1
C ··· H / H ··· C	29.6
N ··· H / H ··· N	1.1
C ··· N / N ··· C	1.7
C ··· C	2.8
