# Supplementary material for: Crystal structure and Hirshfeld analysis of 2-[bis­(1-methyl-1H-indol-3-yl)meth­yl]benzoic acid
Source: Acta Crystallogr E Crystallogr Commun. 2018 Oct 16;74(Pt 11):1580–3. doi: 10.1107/S2056989018014160 (PMC6218917; doi:10.1107/S2056989018014160)
Supplement: Supplementary file 4 [file e-74-01580-sup5.pdf]

Supplementary material

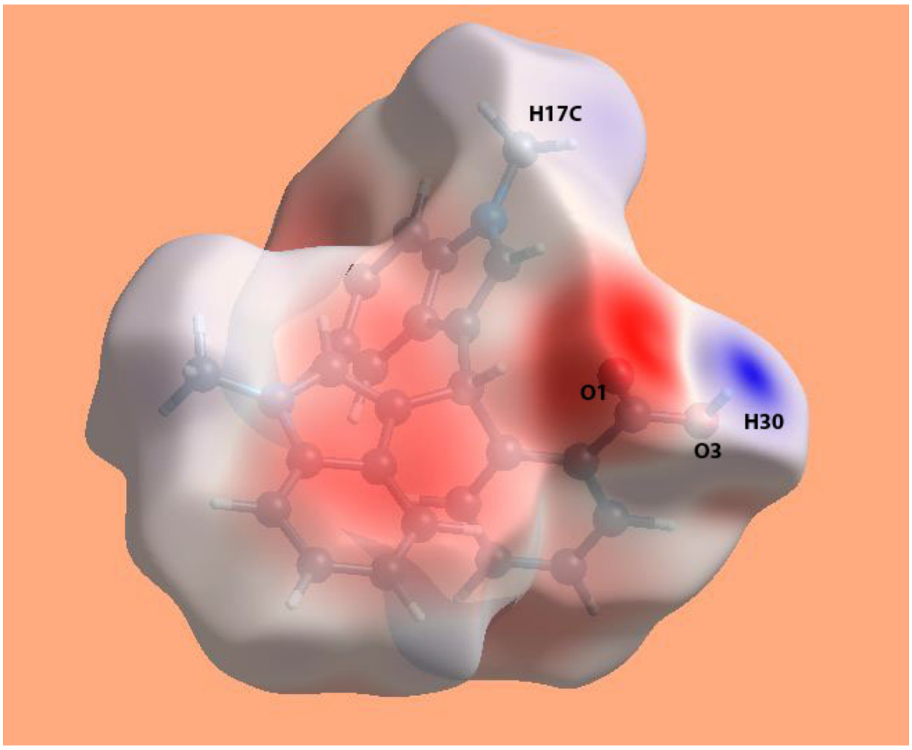

The views of the Hirshfeld surface of the title compound mapped over the electrostatic potential in the range ±0.046 au.

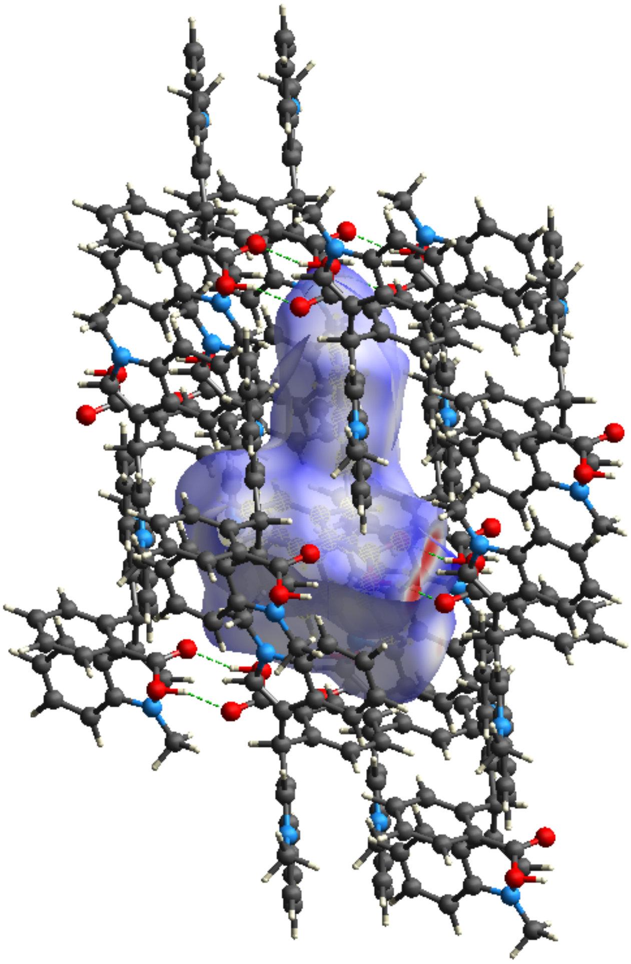

A view of the Hirshfeld surface of the title compound mapped over d_{norm} in the range -0.6842 to +1.4524 au. highlighting C···H/H···C contact by green dashed line.
